# Biodegradation of aflatoxin in dried figs

**DOI:** 10.1007/s11274-025-04466-9

**Published:** 2025-07-08

**Authors:** Senem Öztürk Köse, Hacı Halil Biyik

**Affiliations:** Science Faculty, Biology Department, Aydın Adnan Menderes University, Aydın, Türkiye

**Keywords:** Aflatoxin B1, Aflatoxin G1, Biodegradation, Dried Fig, HPLC

## Abstract

This study sought to ascertain the biodegradation of aflatoxin by adapted microorganisms extracted from dried figs containing aflatoxin, and by products of this biodegradation. Adapted microorganisms were isolated from dried figs containing aflatoxin and were identified morphologically/molecularly. A preliminary assessment was conducted using coumarin medium to evaluate the aflatoxin biodegradation capabilities of microorganisms. Aflatoxin biodegradation was performed with *Bacillus cereus* HBB532, *Bacillus safensis* HBB526, *Priestia megaterium* HBB522, *Zygosaccharomyces rouxii* HBB542 and *Zygosaccharomyces pseudorouxii* HBB545. Microorganism cultures and supernatants were examined separately in terms of biodegradation. Aflatoxin levels were determined by high-pressure liquid chromatography (HPLC). Bacteria reduced aflatoxin B1, aflatoxin G1, and total aflatoxin in dried figs by more than 50%, while yeasts showed limited biodegradation activity. QTOF-LC/MS analyses were performed to determine the products formed after aflatoxin biodegradation. As a result of biodegradation, 22 degradation products were screened and identified. Sugar amounts in dried figs were measured during the biodegradation of aflatoxin and a 25% decrease in sugar levels was detected after the process. This study is the first aflatoxin biodegradation study using dried figs as a matrix. The study results pave the way for sustainable solutions to reduce food waste and increase food security.

## Introduction

Dried figs hold significant economic value for Türkiye. Türkiye is the leading global producer of fresh and dried figs (Nazilli Chamber of Commerce 2019). Aflatoxin production in dried figs presents a concern for food safety and human health, resulting in economic detriment (Karaca et al. 2010).

Aflatoxins are very poisonous mycotoxins generated as secondary metabolites by molds belonging to the Aspergillaceae family (Javanmardi et al. 2020). The initial mold recognized for aflatoxin production was *Aspergillus flavus*. In 1960, it was revealed that *Aspergillus flavus* generated aflatoxin, leading to aflatoxicosis, which resulted in the mortality of numerous agricultural animals in England. The “A” in aflatoxin denotes the Aspergillus genus, while “fla” signifies the flavus species (Fletcher and Blaney 2016). While it is established that aflatoxins are predominantly synthesized by *Aspergillus flavus*, *Aspergillus parasiticus*, and *Aspergillus nomius*, research has indicated that *Aspergillus bombycis*, *Aspergillus ochraceoroseus*, and *Aspergillus pseudotamari* also generate aflatoxin (Prettl et al. 2017). While *Aspergillus flavus* is typically attributed to aflatoxin production, research indicates that 40–50% of *Aspergillus flavus* strains may create aflatoxin, whereas 90% of *Aspergillus parasiticus* strains possess this capability. Aflatoxins have two structural components: a fundamental difuran ring and a coumarin moiety, which confer their poisonous characteristics. The interaction between these two structures amplifies toxicity (Wang et al. 2022). AFB1 exhibits the greatest toxicity. The biodegradation of aflatoxin is an eco-friendly and effective biotechnological process that seeks to alter the structure of poisons into other derivatives. Metabolic pathways and enzyme systems of various microbes are employed to modify the actions of toxins. Microbial transformation technology seeks to identify particular strains that effectively degrade aflatoxin without generating additional hazardous byproducts (Song et al. 2022). Biological agents and enzymes exert minimal influence on the sensory attributes and nutritional quality of food relative to chemical agents, rendering them safer for usage (Loi et al. 2017). Numerous microorganisms capable of degrading aflatoxin have been found (Verheecke et al. 2016). Numerous studies have also indicated that Lactobacillus and yeast species bind to and absorb aflatoxin in food (Luo et al. 2020).

The biodegradation of aflatoxins with enzymes is achieved by biotransformation of aflatoxin into different subunits. Enzymes can be extracted from their sources and utilized in aflatoxin biodegradation; however, as each enzyme possesses distinct operational parameters, the optimization of these circumstances is crucial (Song et al. 2022). The enzymes used and identified for aflatoxin degradation are reductase, oxidase, laccase and peroxidase (Adobe et al. 2017). Modern developments in nanotechnology have also yielded improvements in enzyme research. Liang et al. (2017) developed a laccase mimic nanoenzyme utilizing Cu^+ 2^ and guanosine monophosphate (GMP), demonstrating that its production is 2400 times less expensive than that of commercial laccase. Given that laccase facilitates aflatoxin breakdown, researchers anticipate the integration of this technology into the industry through nanoenzyme studies, which represent an innovative approach owing to their cost-effectiveness, economic viability, and applicability in food processing.

Research has been performed on molds, yeasts, and algae about the breakdown of aflatoxin by eukaryotic organisms. Research on yeasts predominantly focuses on *Saccharomyces cerevisiae*, which is recognized for its ability to reduce aflatoxin through binding or absorption, rather than destruction (Adebo et al. 2017). Research on aflatoxin degradation has been performed using various mold species from the Ascomycota, Basidiomycota, and Zygomycota phyla. Not all Ascomycota members, including aflatoxin-producing *Aspergillus* species, are toxigenic molds. Research indicates that *Aspergillus niger* is capable of degrading aflatoxins (Qui et al. 2021).

A significant amount of dried figs contaminated with aflatoxin are annually disposed of in Türkiye to ensure food safety. The main objective of this study is to carry out the biodegradation of aflatoxin in dried figs using microorganisms isolated from aflatoxin-contaminated dried figs. Additionally, the identification of the products formed as a result of aflatoxin degradation is another goal of this research. Investigating the biodegradation process is essential for converting these products into new raw materials for various uses.

## Materials and methods

### Isolation and identification of adapted microorganisms from aflatoxin contaminated dried figs

Aflatoxin-containing dried figs, totaling 50 g, were mixed with 450 mL of physiological salin under aseptic conditation until a homogeneous mixture was obtained. This homogenate were serially diluted with physiological saline (0,85%) and inoculated onto nutrient agar (NA), Dichloran-Rose Bengal Chloramphenicol Agar (DRBC), Yeast Extract Peptone Dextrose agar (YPD) and Mannitol-Egg-Yolk-Polymyxine Agar (MYP) using the spreading technique and incubated at appropriate temperatures. Pure colonies were suspended in sterile distilled water, transferred into eppendorf tubes, and stored at -20 °C for subsequent DNA isolation. DNA isolation from microorganisms was performed using a commercial kit (GENEMARK Bacteria/Plant Genomic DNA Purification Kit). In the polymerase chain reaction (PCR), the universal 16 S primers 27 F and 1492R (0.3 µl each) were used for bacterial isolates, while the 18 S primers ITS1 (5´-TCCGTAGGTGAACCTGCGG-3´) and ITS4 (5´-TCCTCCGCTTATTGATATGC-3´) were used for fungal isolates at 0.3 µl each for molds and 1.6 µl each for yeasts. PCR amplicons were sequenced by TrioGen as a service purchase. The analyzed DNA sequence results were compared with the data in GenBank using the nBLAST program (https://blast.ncbi.nlm.nih.gov/) and molecular identification was performed.

### Preliminary screening of microorganisms in coumarin medium

Three distinct coumarin media were produced for preliminary screening experiments by changes of reference publications. The assessment of adaptable microorganisms cultivated in liquid coumarin media was performed first. Secondly, carbon and glucose sources in nutrient agar, YPD agar, and malt extract agar were removed and replaced with a coumarin component. In the third step, a solid coumarin medium was created by incorporating 15 g/l of agar into the liquid coumarin medium. Microorganism inoculation was conducted as a negative control group by streaking on solid medium devoid of coumarin, maintaining identical conditions (Guan et al. 2008; Xia et al. 2017). *Aspergillus niger*, *Bacillus subtilis* ATCC 6633 and *Saccharomyces cerevisiae*, which are microorganisms that have been proven to biodegrade aflatoxin in previous scientific studies, were used as positive controls in the medium (Afsharmanesh et al. 2018; Fang et al. 2020; Liu et al. 2021).

### Growth rate and aflatoxin biodegradation medium optimization

A Box-Behnken design created in MINITAB was applied to optimize the growth conditions of *B. cereus* HBB532. Cultures were incubated in media adjusted to pH 5.6 and 7, at 30 °C, 35 °C, and 40 °C, with inoculum volumes of 0.5, 1, and 1.5 mL. Based on the analysis, pH and temperature were identified as significant factors influencing growth, whereas inoculum size exhibited a lesser effect. Therefore, in subsequent optimization experiments with other microorganisms, inoculum volume was fixed at 1 mL, and only pH and temperature were varied. In these experiments, cultures containing 0.5 McFarland standard bacterial suspensions or 10⁷ yeast cells were inoculated into NB or YEPD media at pH 5.6 and 7. Each strain was incubated at 3 different temperatures (bacteria: 30 °C, 35 °C, 40 °C/yeast: 27℃, 30℃, 35℃). Microorganism growth was monitored as turbidity until the death phase. Microorganism growth was controlled with a spectrophotometer (PerkinElmer UV-VIS) using 600 nm optical density. *Bacillus subtilis* ATCC 6633 and *Saccharomyces cerevisiae* ATCC 9763 were used as control cultures.

The aflatoxin biodegradation investigation utilized a modified approach by Adebo et al. (2017) to ascertain the volumetric ratio of microorganisms to be inoculated onto dried figs. Each bacterial culture was prepared as 0.5 and 1 McFarland, and each yeast culture was prepared as 1 × 10^9^ and 1 × 10^7^ cells. Fresh cultures were added to the aflatoxin standard (30 ng/g) (9V/1V) and incubated in a shaking incubator at 200 rpm. The absorbances of bacterial and yeast cells at specific time intervals in the spectrophotometer (600 nm) were assessed as a kinetic measurement of aflatoxin biodegradation.

Microorganism supernatants were made following the methodology of Liang Xu (2017). Five and nine milliliters of each microorganism supernatant were combined with one milliliter of aflatoxin standard (10 ng/g) and incubated. Aflatoxin levels were determined using HPLC analysis at specific intervals.

### Determination of aflatoxin biodegradation amounts

The biodegradation levels of microorganisms and supernatants in aflatoxin-contaminated dried figs were assessed using HPLC analysis (AOAC 999.07). In aflatoxin analysis, the mobile phase consisted of water/methanol/acetonitrile (6v + 2v + 3v), with a flow rate of 1 mL/min. Chromatographic separation was performed using a C18 ODS3 column, and detection was carried out with a fluorescence detector set at an excitation wavelength of 360 nm and an emission wavelength of 440 nm. Dried figs containing aflatoxin were diluted to one-third and homogenized under aseptic conditions for the biodegradation analysis. Cultures and supernatants containing 1 McFarland bacteria / 10^9^ yeast were prepared in 450 mL and 50 mL of dried fig homogenate containing aflatoxin was added. The mixture was incubated in a shaking incubator. Aflatoxin degradation by both bacterial and yeast cultures, as well as by bacterial supernatants, was measured on days 0, 5, and 11 of incubation. For yeast supernatants, the analysis was conducted on days 0 and 14 of incubation. Two different control groups were established by incorporating 450 mL of sterile distilled water into 50 mL of aflatoxin-laden dried fig homogenate. According to the optimization study results, control group 1 was incubated at pH 6 and 35 °C, and control group 2 was incubated at pH 7 and 30 °C.

### Determination of aflatoxin biodegradation products

The identification of biodegradation products was conducted using QTOF-LC/MS studies (Suresh et al. 2020). An ion library was constructed based on a literature review, and these ions were screened to identify aflatoxin biodegradation products. Aflatoxin-free dried fig sample served as a negative control. In aflatoxin degradation production analysis, chromatographic separation was performed using a HPLC Agilent 1260 Infinity series (Agilent Technologies, Santa Clara, CA, USA) instrument with a Poroshell 120 EC-C18 (3.0 × 50 mm, 2.7 μm particle size) column. The mobile phase system was constructed using a gradient elution of 0.1% formic acid in water (A) and acetonitrile (B) as follows: 0–0.5 min, 10% B; 0.5–5 min, 70% B; 5–7 min, 95% B; 7–10 min, 95% B; 10–15 min, 10% B. The column oven was maintained at 35 ° C. The injected sample volume was 10 µL, and the flow rate was selected as 0.5 mL/min.

MS analysis was performed using an Agilent 6550 iFunnel high resolution Accurate Mass Q-TOF / MS equipped with the Agilent Dual Jet Stream electrospray ionization (Dual AJS ESI) interface operating in positive ion in the following cases: drying gas flow, 14.0 L/min; nebulizer pressure, 35 psi; gas drying temperature, 290 ° C; sheath gas temperature, 400 ° C; sheath gas flow, nitrogen 12 L / min. MS/MS spectra were collected with collision energies of 10 eV. The scanning range m z was selected from 40 to 400. The acquisition was controlled by Agilent MassHunter Acquisition Software Ver. A.09.00 and the data were processed with MassHunter Qualitative Software Ver. B.07.00.

### Sugar analyses

In aflatoxin biodegradation studies, sugar amounts from dried fig samples were analyzed by the Luff-Schoorl Method on the first and last days of incubation.

### Statistical analyses

The data obtained as a result of the study were evaluated by applying statistical analyses. The dependent variable in the study is the amount of aflatoxin biodegradation in dried figs, and the independent variable is the microorganisms used. The relationship between dependent and independent variables was evaluated by applying an ANOVA test (*n* = 3).

## Results

### Preliminary screening of coumarin medium

**Fig. 1:**
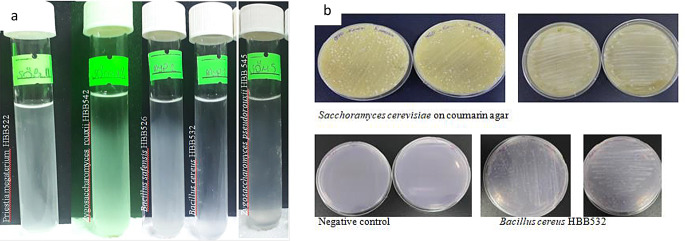
Microbial growth in the presence of coumarin. **a** Microorganisms exhibiting growth in liquid coumarin medium. White turbidity in the tubes indicates microbial proliferation. Strains that showed growth in at least two out of three replicates were re-cultivated on solid coumarin medium for confirmation; **b** Microbial growth observed on solid coumarin medium for *B. cereus* HBB532, positive control *Saccharomyces cerevisiae*, and negative control petri dishes. Growth was observed in the positive control group (*Saccharomyces cerevisiae*), while no microbial development was detected in the negative control group

A total of six bacterial strains, two yeast strains, and two fungal strains were isolated from aflatoxin-contaminated dried figs and subsequently identified based on morphological and molecular characteristics. Among the isolates, *B. safensis* HBB526, *P. megaterium* HBB522, *B. cereus* HBB532, *Z. rouxii* HBB542, and *Z. pseudorouxii* HBB545 demonstrated the ability to grow in liquid coumarin medium. These isolates, along with the reference strains (*Aspergillus niger*, *Bacillus subtilis* YL336, *Bacillus subtilis* ATCC 6633, and *Saccharomyces cerevisiae*), were also cultivated on solid coumarin medium, where growth was observed. In contrast, no microbial growth was detected in the negative control petri dishes. Images of studies conducted in liquid and solid coumarin media are presented in Fig. 1a and b.

### Optimization of aflatoxin biodegradation medium

**Fig. 2:**
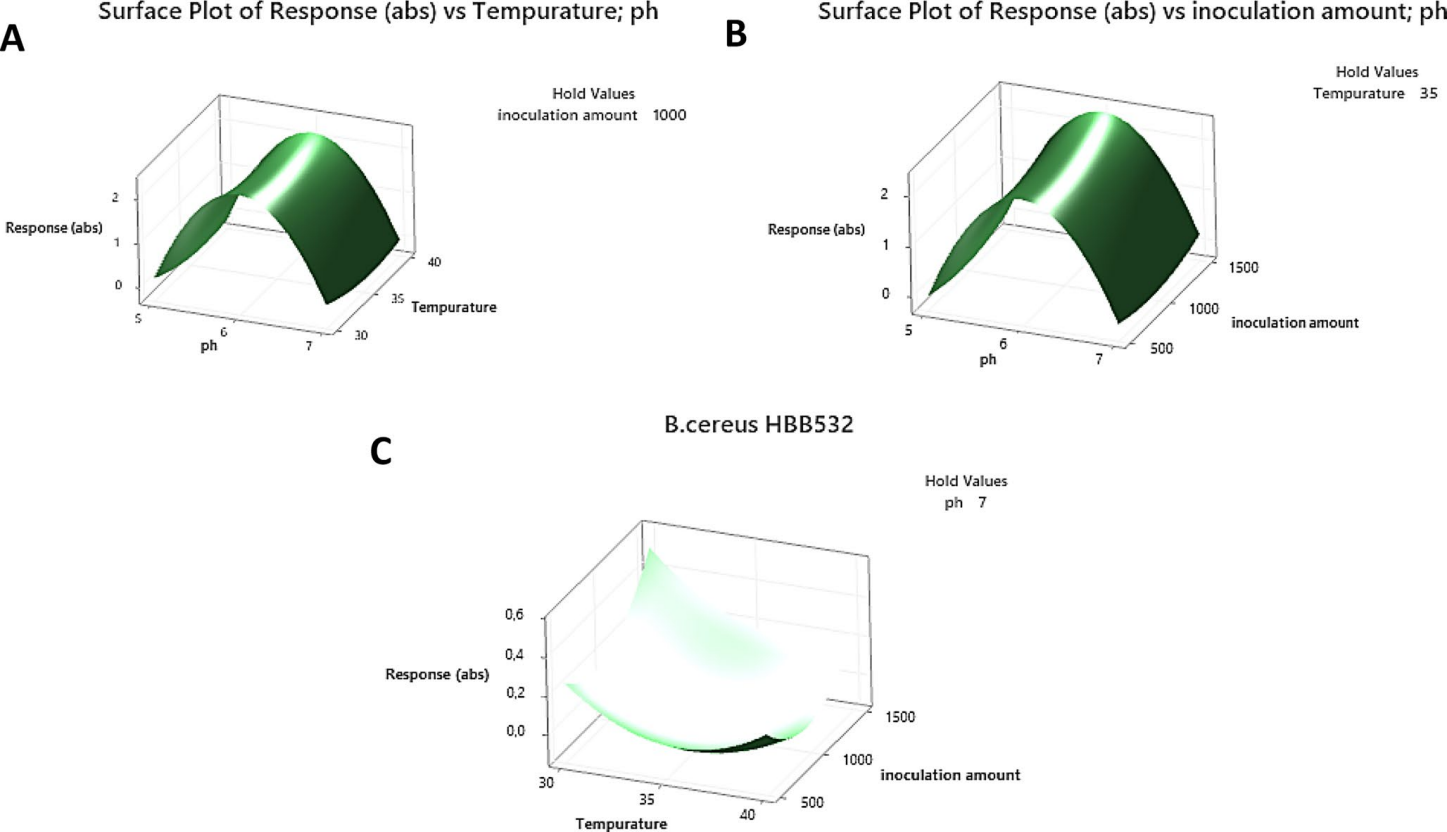
Response Surface Plots for *B. cereus* HBB532. **a** The highest absorbance for *B. cereus* HBB532 was reached at pH 6 and temperature around 35 °C. Biological activity decreases in acidic environments and high temperatures; **b** Under constant temperature (35 °C), absorbance value is low in acidic environments (pH5) and low inoculation amounts (500 µl). A slight decrease in absorbance is also observed when the inoculation amount is very high (1500 µl); **c** A slight increase in absorbance is observed in the temperature range of 30–35 °C and low inoculation amounts. As temperature and inoculation amount increase, a significant decrease in absorbance occurs

It was determined that *B. cereus* HBB532 had the best growth curve at pH 6, 35℃, *P. megaterium* HBB522 and *B. safensis* HBB526 at pH 6, 30℃, *Z. rouxii* HBB542 and *Z. pseudorouxii* HBB 545 at pH 7 and 30℃. No microorganism growth was detected in the medium with a pH of 5. The positive control *Bacillus subtilis* ATCC6633 showed the optimal growth curve at pH 7 and 30 °C, while the *Saccharomyces cerevisiae* ATCC 9763 thrived at pH 6 and 30 °C The optimal conditions were employed for aflatoxin biodegradation in dried fig. The response surface graphs for the optimization of *B. cereus* HBB532 growth conditions using the Box-Behnken design (pH × temperature, pH × inoculum size, and temperature × inoculum size) are presented in Fig. 2a and b, and 2c.

Consequently, spectrophotometric studies indicated that a 1 McFarland inoculation at a 1 V + 9V volumetric ratio is suitable for bacterial cultures, whereas a 10^9^ cells/mL 1 V + 9V volumetric ratio is appropriate for yeasts. Table 1 presents the findings of the investigation aimed at quantifying the inoculation levels in microorganism cultures.

**Table 1:** Aflatoxin biodegradation times of microorganism cultures inoculated with aflatoxin standard at certain rates

Microorganism Name	Inoculation amount	Biodegredation time (hours-day)
*B. cereus* HBB532	0,5 Mcfarland	140 h
	1 McFarland	120 h
*B. safensis* HBB526 *P. megaterium* HBB522 *B. subtilis* ATCC6633	0,5 Mcfarland	144 h
	1 McFarland	72 h
*Z. pseudorouxii* HBB545	10^7^ cell/mL	13 day
	10^9^ cell/mL	11 day
*Z. rouxii* HBB542	10^7^ cell/mL	96 h
	10^9^ cell/mL	
*Saccharomyces cerevisiae* ATCC9763	10^7^ cell/mL	120 h
	10^9^ cell/mL	72 h

Bacterial supernatants (1 V + 9 V) demonstrated effective biodegradation of aflatoxins. The supernatants of yeast isolates exhibited no degradation of aflatoxin. The results of the optimization study for aflatoxin biodegradation of bacterial supernatants are given in Table 2.

**Table 2:** The results of the optimization study for aflatoxin biodegradation of bacterial supernatants

Microorganism Name-Supernatant amount	Aflatoxin Biodegradation Rates (%)				
	AFB1	AFB2	AFG1	AFG2	Total AF
*B. cereus* HBB532–10x	62	58	100	100	78
*B. cereus* HBB532–5x	57	30	100	100	69
*B. safensis* HBB526–10x	71	65	100	100	82
*B. safensis* HBB526- 5x	56	26	100	100	68
*P. megaterium* HBB522–10x	69	64	100	100	81
*P. megaterium* HBB522–5x	63	45	100	100	74

### Aflatoxin biodegradation

In a medium containing aflatoxin from dried figs;

*B. cereus* HBB532 culture degraded AFB1 by 51%, AFB2 by 44%, AFG1 by 38%, AFG2 by 35%, and total aflatoxin (TAF) by 45%. Its supernatant degraded AFB1 by 17%, AFB2 by 8%, AFG1 by 5%, and TAF by 14%.

*B. safensis* HBB5262 degraded AFB1 by 51%, AFB2 by 41%, AFG1 by 31%, AFG2 by 29% and TAF by 42%. The supernatant was unable to degrade AFB2 and AFG2, while it degraded AFB1 by 5%, AFG1 by 69%, and TAF by 22%.

*P. megaterium* HBB522 degraded AFB1 by 59%, AFB2 by 100%, AFG1 by 70%, AFG2 by 100%, and TAF by 63%. The bacterial supernatant was ineffective in degrading AFB2 and AFG2, although it reduced AFB1 by 24%, AFG1 by 68%, and TAF by 38%.

The *Z. rouxii* HBB542 culture at pH 7, 30 °C, and incubated for 11 days achieved a biodegradation of 5% AFB1 and 7% AFB2 aflatoxins. No deterioration was seen for AFG1, AFG2, and TAF. No deterioration was detected in the investigations performed with this microorganism supernatant.

No biodegradation of aflatoxin occurred after 11 days of incubation with *Z. pseudorouxii* HBB545 culture. The supernatant degraded AFB1 by 12%, AFB2 by 36%, AFG1 by 17%, AFG2 by 45%, and TAF by 15%.

When the degradation results of the control 1 group were examined, it was seen that AFB1 was degraded by 59%, AFB2 by 100%, AFG1 by 65%, AFG2 by 100%, and TAF by 62%.

When the degradation results of the control 2 group were examined, it was seen that AFB2 and AFG2 were not degraded, while AFB1 was degraded by 27%, AFG1 by 9%, and TAF by 25%.

**Fig. 3:**
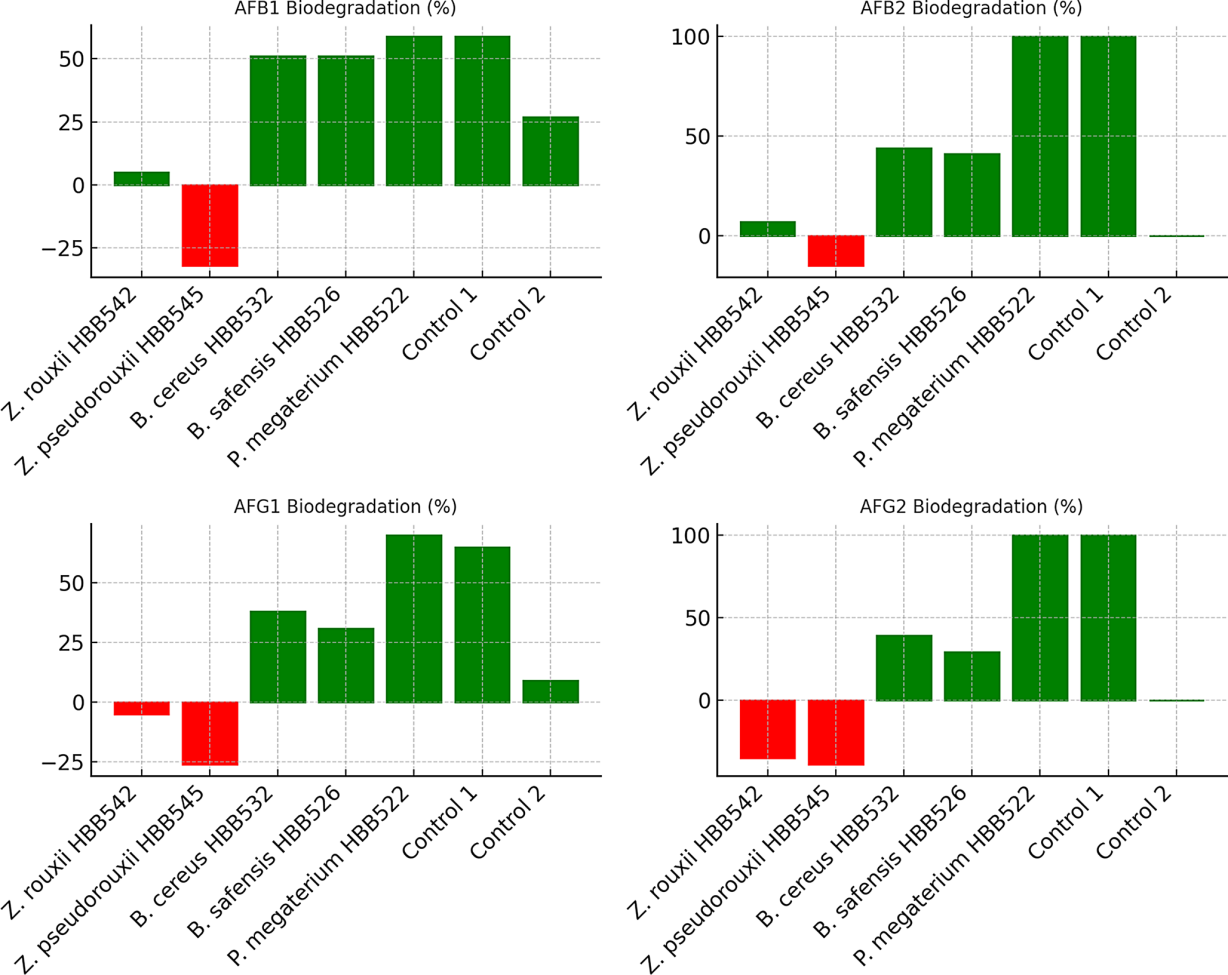
Biodegradation percentages of individual aflatoxins (AFB1, AFB2, AFG1, and AFG2) by different microorganisms (*Z. rouxii* HBB542, *Z. pseudorouxii* HBB545, *B. cereus* HBB532, *B. safensis* HBB526, *P. megaterium* HBB522) and control groups. Positive biodegradation percentages (indicating toxin reduction) are shown in green, whereas negative values (indicating toxin increase) are shown in red. *P. megaterium* HBB522 demonstrated the highest biodegradation efficiency, especially achieving 100% degradation for AFB2 and AFG2

**Fig. 4:**
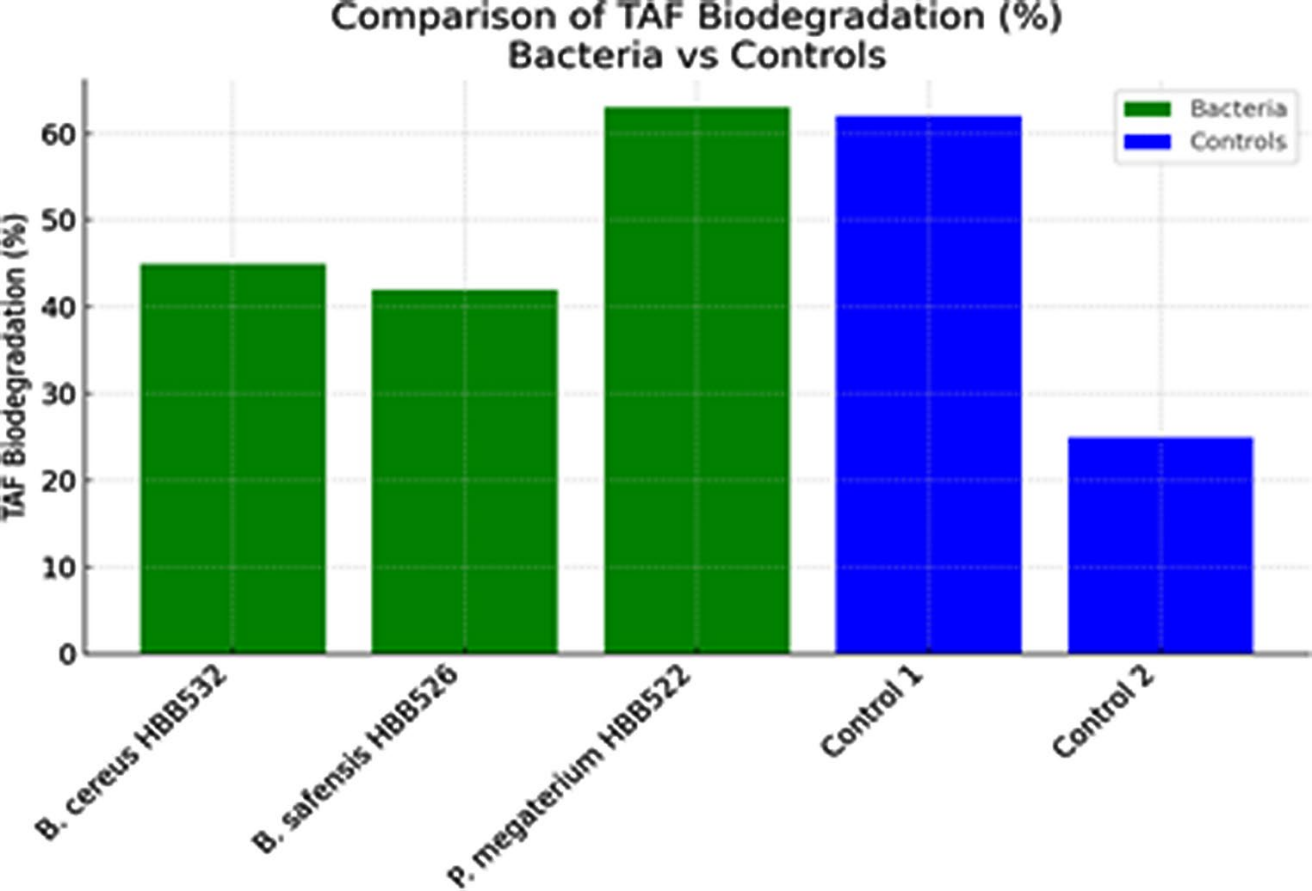
Comparison of overall TAF (Total Aflatoxin) biodegradation percentages between bacterial isolates (*B. cereus* HBB532, *B. safensis* HBB526, *P. megaterium* HBB522) and control groups. *P. megaterium* HBB522 achieved the highest TAF biodegradation (63%), followed closely by Control 1 (62%). Control 2 showed significantly lower biodegradation (25%), emphasizing the contribution of bacterial activity to aflatoxin reduction

**Fig. 5:**
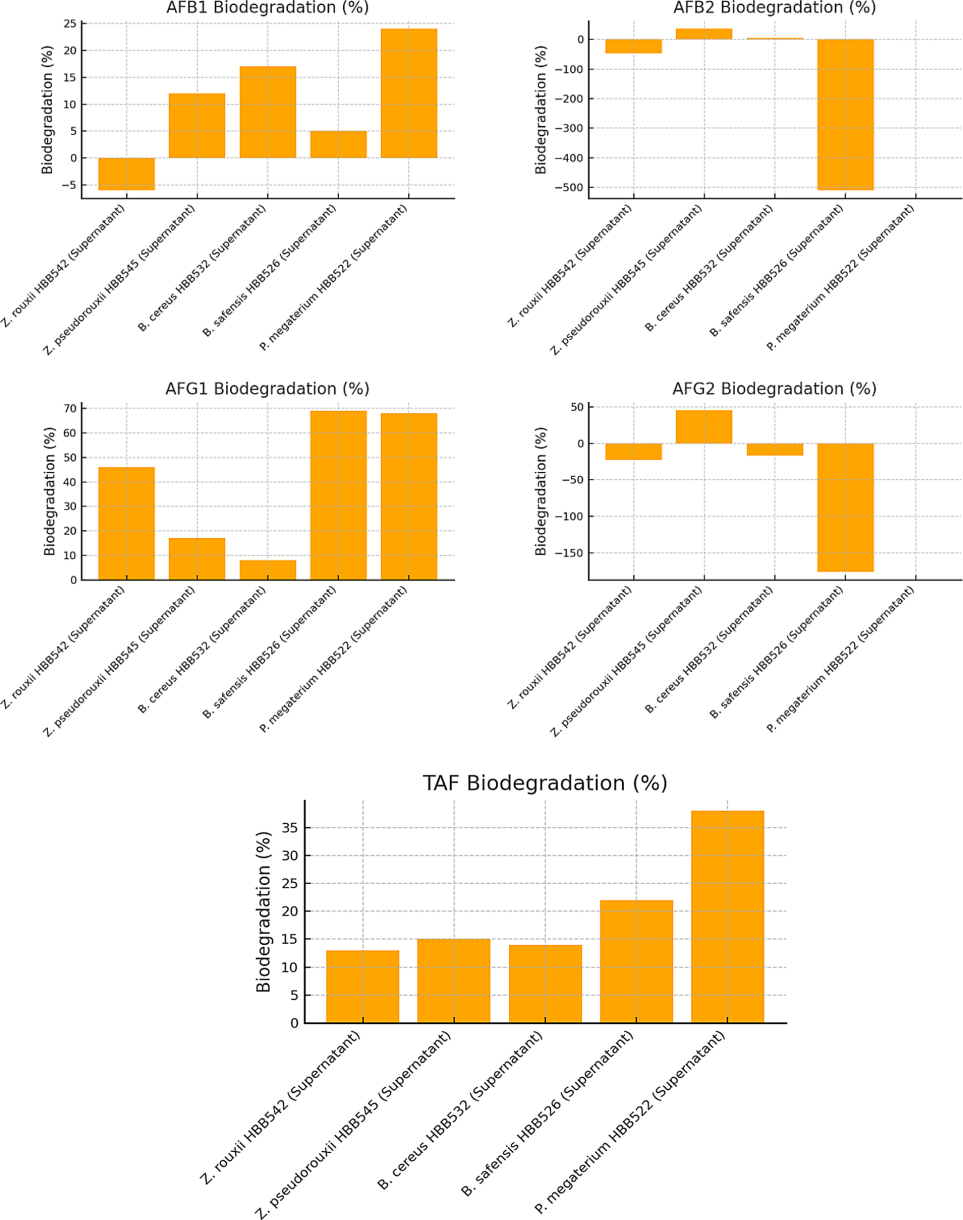
Aflatoxin (AFB1, AFB2, AFG1, AFG2) and TAF biodegradation percentages by different microbial supernatants(%). The biodegradation effects of culture supernatants from *Z. rouxii* HBB542, *Z. pseudorouxii* HBB545, *B. cereus* HBB532, *B. safensis* HBB526, and *P. megaterium* HBB522 on AFB1, AFB2, AFG1, AFG2, and TAF were comparatively evaluated. Biodegradation percentages for each aflatoxin type are presented in separate graphs. Among the tested strains, *P. megaterium* HBB522 supernatant exhibited the highest overall biodegradation activity

Detailed HPLC data on aflatoxin biodegradation in dried figs are presented in Table 3. The corresponding graphical representations are shown in Figs. 3 and 4. HPLC results from aflatoxin degradation studies using the supernatants of microorganisms are summarized in Table 4, with the related graph provided in Fig. 5.

**Table 3:** Biodegradation of aflatoxin in dried figs by microorganisms HPLC data

Organism	Toxin	Day 0 (ng/g)	Day 5 (ng/g)	Daay 11 (ng/g)	Biodegredation (%)
*Z. rouxii* HBB542	AFB1	14,98	17,06	14,3	5
	AFB2	1,82	2,11	1,69	7
	AFG1	2,43	3,01	2,54	-5
	AFG2	0,34	0,55	0,46	-35
	**TAF**	19,57	22,74	18,99	3
*Z. pseudorouxii* HBB545	AFB1	14,32	20,74	18,85	-32
	AFB2	1,76	2,04	2,02	-15
	AFG1	2,38	3,19	2,99	-26
	AFG2	0,41	0,51	0,57	-39
	**TAF**	18,87	26,49	24,44	-30
*B. cereus* HBB532	AFB1	49,9	35,87	24,3	51
	AFB2	6,96	3,49	3,87	44
	AFG1	38,74	31,12	24,07	38
	AFG2	3,4	1,36	2,09	39
	**TAF**	98,5	71,84	54,32	45
*B. safensis* HBB526	AFB1	47,47	35,09	23,36	51
	AFB2	6,13	2,78	3,6	41
	AFG1	36,36	36,61	25,21	31
	AFG2	2,71	1,06	1,93	29
	**TAF**	92,67	75,53	54,11	42
*P. megaterium* HBB522	AFB1	132,55	82,45	54,72	59
	AFB2	6,67	11,04	0	100
	AFG1	30,25	23,69	9,06	70
	AFG2	2,18	3,68	0	100
	**TAF**	171,65	120,85	63,78	63
Control 1	AFB1	160	86,57	64,83	59
	AFB2	5,76	7,71	0	100
	AFG1	46,16	33,19	15,98	65
	AFG2	2,21	2,6	0	100
	**TAF**	214	130,14	80,81	62
Control 2	AFB1	113,2	102,91	82,38	27
	AFB2	0	0	0,38	0
	AFG1	17,93	21,51	16,4	9
	AFG2	0	0	0	0
	**TAF**	131,15	124,42	98,81	25

**Table 4:** Biodegradation of aflatoxin in dried figs by microorganisms supernatant HPLC data

(ng/g)	0 Day	5 Day	11 Day	14 Day	Biodegradation (%)
***Z. rouxii*** **HBB542**					
AFB1	34,13	-	-	36,26	-6
AFB2	1,42	-	-	2,07	-46
AFG1	23,8	-	-	12,79	46
AFG2	0,92	-	-	1,13	-23
TAF	60,27	-	-	52,23	13
***Z. pseudorouxii*** **HBB545**					
AFB1	33,91	-	-	29,91	12
AFB2	2,11	-	-	1,36	36
AFG1	23,39	-	-	19,37	17
AFG2	1,38	-	-	0,76	45
TAF	60,8	-	-	51,4	15
***B. cereus*** **HBB532**					
AFB1	16,47	14,91	13,72	-	17
AFB2	2	1,71	1,91	-	5
AFG1	2,85	2,65	2,63	-	8
AFG2	0,53	0,46	0,62	-	-17
TAF	21,85	19,74	18,88	-	14
***B. safensis*** **HBB526**					
AFB1	32,72	41,96	31,17	-	5
AFB2	0,51	3,98	3,11	-	-510
AFG1	19,69	23,66	6,09	-	69
AFG2	0,41	2,34	1,13	-	-176
TAF	53,31	71,94	41,5	-	22
***P. megaterium*** **HBB522**					
AFB1	38,04	36,19	28,89	-	24
AFB2	2,51	2,73	2,51	-	0
AFG1	23,55	18,94	7,62	-	68
AFG2	1,33	1,2	1,33	-	0
TAF	65,44	59,06	40,36	-	38

### Identification of biodegradation products

**Fig. 6:**
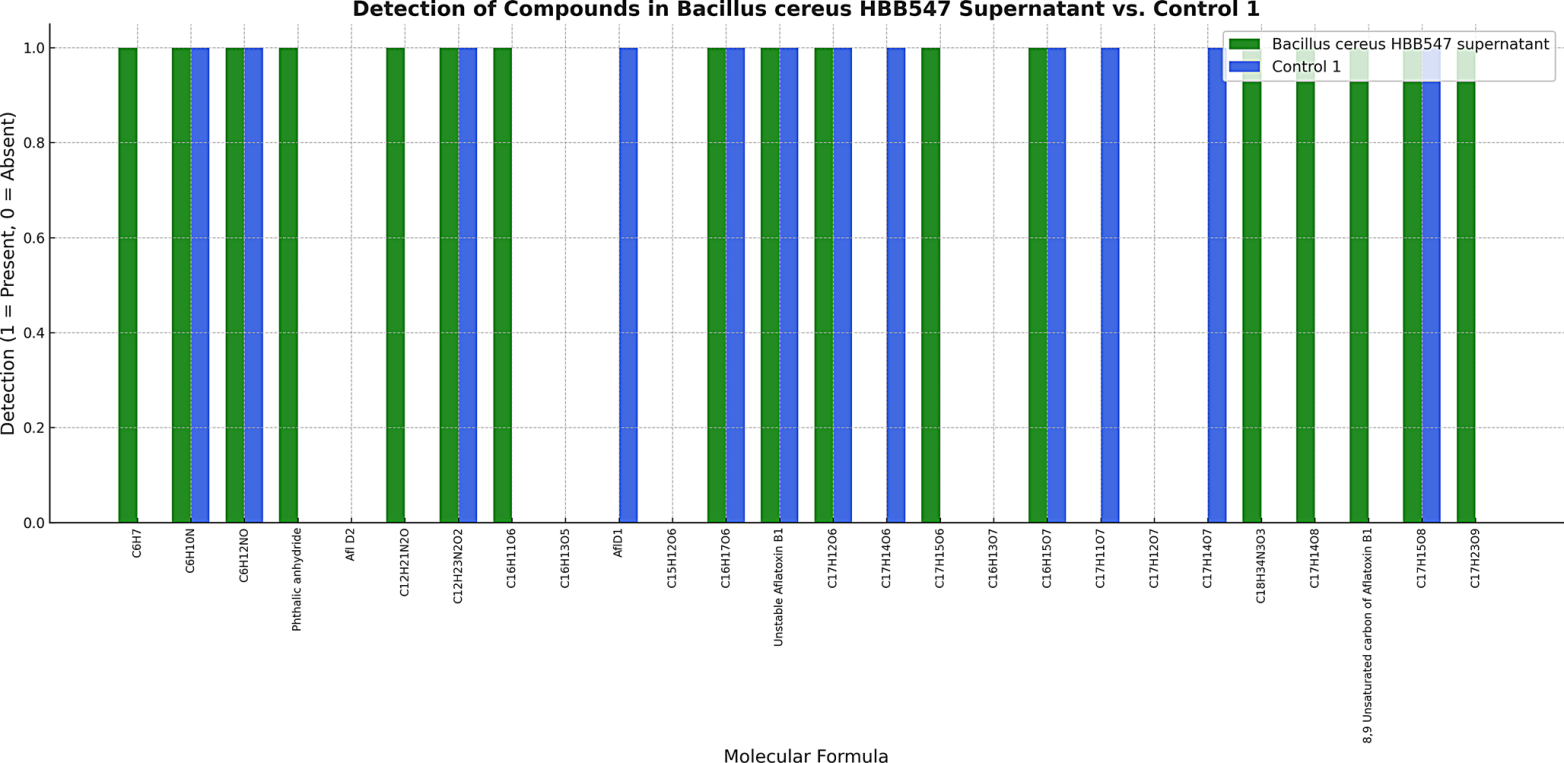
Detection profile of chemical compounds based on molecular formulae in *B. cereus* HBB547 supernatant and Control 1 samples. Bars represent the presence (1) or absence (0) of specific compounds. Green bars correspond to detections by *B. cereus* HBB547 supernatant, while blue bars correspond to detections in the Control 1 sample, highlighting differential metabolic or degradation activities. Note: The Y-axis represents binary detection status (presence = 1; absence = 0) and does not reflect ion count, intensity, or relative abundance

**Fig. 7:**
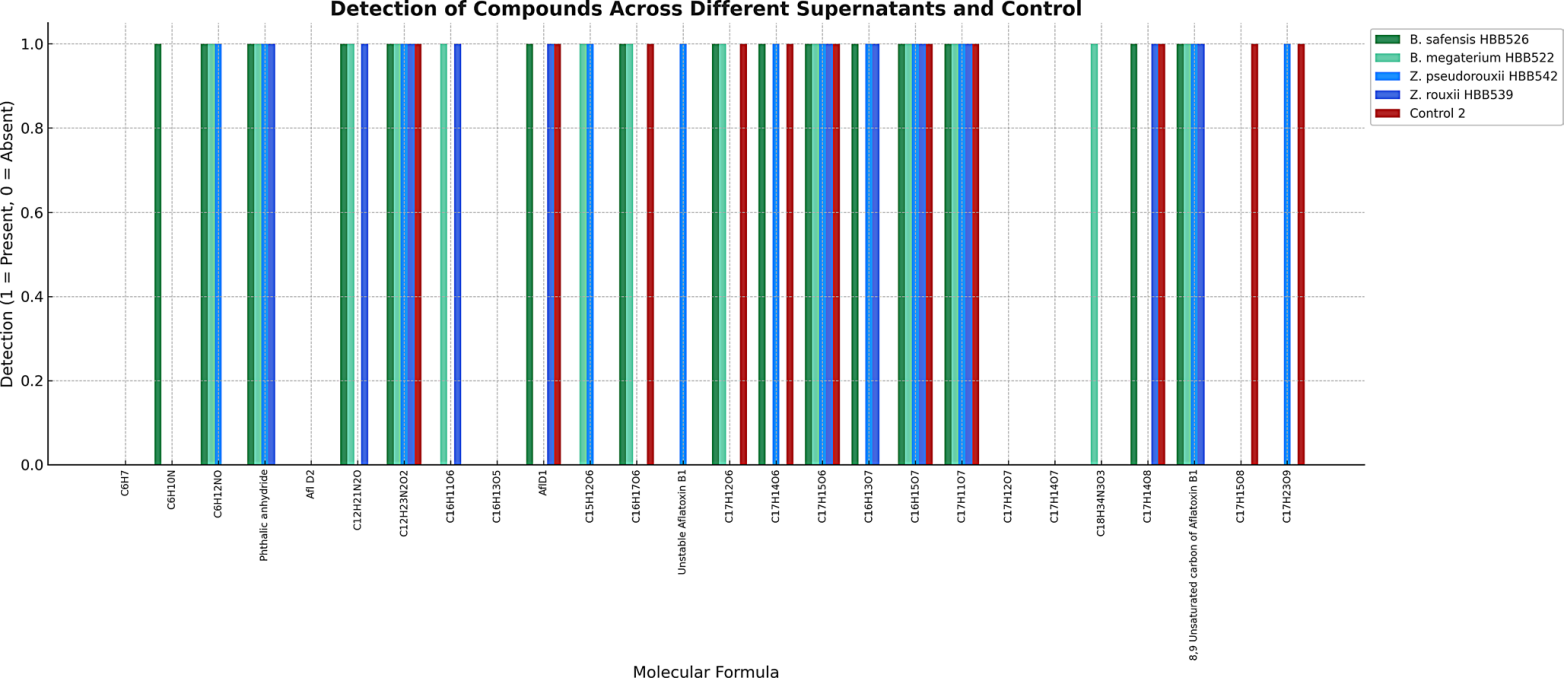
Comparative detection profiles of chemical compounds in the supernatants of *B. safensis* HBB526, *Bacillus megaterium* HBB522, *Z. pseudorouxii* HBB542, *Z. rouxii* HBB539, and Control 2. The presence (scored as 1) or absence (scored as 0) of molecular species was determined based on their molecular formulae. Distinct metabolic and degradative capacities were observed among bacterial and yeast-derived supernatants, with several compounds exclusively detected or absent in specific groups, indicating differential enzymatic activities and biotransformation potential relative to the control. Note: The Y-axis represents binary detection status (presence = 1; absence = 0) and does not reflect ion count, intensity, or relative abundance

The compounds resulting from the biodegradation of aflatoxin were identified by QTOF-LC/MS analysis. Since QTOF-LC/MS tests were performed on dried fig matrix containing aflatoxin, several ions originating from the matrix were also discovered using the same methods. Therefore, a library was created with 22 ions determined by scanning previous studies in which aflatoxin degradation products were detected, and these ions were searched. In our study results, aflatoxin D1 and C_16_H_17_O_6_ (m/z 305.15) were detected in all biodegradation reactions using microorganism cultures. Analysis of the supernatant degradation products revealed the presence of C_12_H_23_N_2_O_2_ (m/z 227.15) and C_16_H_15_O_7_ (m/z 319.12) as biodegradation products produced by each microorganism. The microorganisms responsible for ion formation due to biodegradation and the corresponding ionic formulae are presented in Figs. 6 and 7. The sugar content in dried figs diminished by 25% following biodegradation.

### Statistical analysis

One-way analysis of variance (ANOVA) was applied to the obtained data. As a result of one-way ANOVA analyses, no statistically significant difference was found between the independent variables (*p* > 0.05).

## Discussion

Given that the coumarin molecule constitutes the primary component of aflatoxin, prior investigations have indicated that strains capable of metabolizing coumarin can also utilize aflatoxin (Guan et al. 2008; Xia et al. 2017). The growth of the positive control group in the coumarin medium shows the accuracy of the method used. In addition, the growth of the test microorganisms in this medium shows that the preliminary studies on aflatoxin biodegradation were successful. In instances where HPLC is unfeasible, the evidence from this investigation corroborates that coumarin medium yields dependable results for aflatoxin biodegradation screenings. Since the optimum growth conditions of microorganisms represent the environmental conditions where biological activity and metabolite production are at their highest, determining these conditions is of great importance for aflatoxin biodegradation. In our study, optimization studies were carried out by taking into account pH and temperature parameters. Successful results were obtained for bacteria under these optimum conditions in aflatoxin biodegradation, but further optimization studies are needed for yeasts. *B. cereus* HBB532 is unable to grow in acidic environments (pH 5), and its growth capability in moderately acidic environments (pH 6) varies depending on the temperature. The optimum growth conditions of pH 7 and 30 °C are typical for many mesophilic bacteria. *B. cereus* is generally mesophilic, growing in the range of 4–55 °C and pH 4.3–9.3. The optimum growth pH range is 6.5–7.5, and the temperature range is 30–40 °C (Le Marc et al. 2021). Studies have shown that the optimal growth conditions of *B. cereus* vary depending on the matrix and temperature range (Ellouze et al. 2021).

When examining the growth conditions of *B. safensis* HBB526 and *P. megaterium* HBB522, no growth was observed in pH 5 and pH 6 at 30 °C. Upon examining growth curves under other conditions, the highest OD600 value in the log phase was obtained at pH 7 and 30 °C. Therefore, pH 7 and 30 °C were chosen as the optimum growth conditions. Studies with *B. safensis* have shown that the effects of pH and temperature on growth result in delayed growth in acidic and alkaline (pH 5–pH 9) environments (Mantea et al. 2025). Optimization studies with *P. megaterium* have shown that this microorganism can grow in a wide range of pH (4–10) and temperature (20–50 °C) conditions. For mesophilic species, pH 7 and 37 °C are considered optimum conditions (Goswami et al. 2018; Mahariawan et al. 2020; Hwang et al. 2022). The optimum growth conditions of microorganisms are variable, depending not only on environmental (physical) factors but also on genetic differences at the species and strain level. In studies focusing on aflatoxin biodegradation, optimization of the culture medium emerges as an important parameter. In various studies conducted with *Bacillus* species, aflatoxin degradation rates at different pH and temperature values have been evaluated, and these rates have been compared with the microorganism’s optimum growth conditions. The results indicate that biodegradation occurs at the highest level under the optimum growth temperature and pH of the microorganism. This suggests that the enzymatic systems involved in aflatoxin degradation also exhibit maximum activity under these conditions (Shu et al. 2018; Xiong et al. 2022).

In optimization studies conducted for aflatoxin biodegradation of microorganisms, 10x culture and supernatant results were more effective than 5x results, supporting a dose-dependent effect. When Table 2 is examined, although AFB1 and AFB2 aflatoxins appear to be more resistant, degradation rates of these toxins are seen in the range of 60–70% in 10-fold supernatant application. In this application, especially aflatoxins AFG1 and AFG2 were completely (100%) degraded. These findings reveal that *Bacillus* supernatants have a high degradation capacity, especially on G group aflatoxins, and this effect increases depending on the supernatant concentration. *Bacillus* species are recognized as biocontrol agents for *A. flavus* and facilitate aflatoxin biodegradation through their bioactive substances or enzymes (Abdel-Sahafi 2018; Pereyra et al. 2019; Einloft et al. 2021; Ren et al. 2020). When the aflatoxin biodegradation results of *B. cereus* HBB532 and *B. safensis* HBB526 are examined, it is seen that bacterial cultures perform degradation at a higher rate than the supernatants. In this situation, aflatoxin biodegradation is attributed to internal enzymes or bioactive components rather than external enzymes. *P. megaterium* includes genes that impede the proliferation of *A. flavus* and peptides that prevent aflatoxin production (Chen et al. 2019; Ahmad et al. 2022). The *P. megaterium* HBB522 culture is the most effective microbe for aflatoxin degradation in both aflatoxin standard and dried fig mediums. In addition, the most effective degradation in dried fig medium was determined with *P. megaterium* HBB522 supernatant. Within the scope of these data, it is seen that *P. megaterium* HBB522 enzyme systems are effective in aflatoxin degradation and it is the microorganism that continues its development best by adapting to dried fig medium. The lactone hydrolase enzyme identified in *Bacillus* species focuses on the lactone ring, initiating the aflatoxin degradation process by disrupting this structure (Wang and Xie 2020). In our study, it was determined that AFG1 and AFG2 were completely degraded by all bacterial supernatants. It is likely that the lactone hydrolase enzymes of these bacteria are effective in the hydrolysis of the AFG1 lactone ring. Although bacterial supernatants are quite successful in standard aflatoxin biodegradation, the low biodegradation in the aflatoxin-containing dried fig medium can be attributed to the matrix effect. There is an adapted microorganism in the dried fig medium. In this competitive medium, the microorganism remains alive and synthesizes components that will degrade aflatoxin and reduces the aflatoxin rate, indicating that the main goal of the study has been achieved. Biodegradation studies utilizing yeast supernatants have revealed that optimizations have performed across various parameters, including temperature, pH, salt concentrations, and metal ion additions. (Rao et al. 2017; Xia et al. 2017; Shu et al. 2018; Suresh et al. 2020; Cai et al. 2020; Zhao et al. 2022). The supernatant of *Z. pesudorouxii* HBB545 was effective in the degradation of aflatoxin in dried figs, although in low amounts. In order to increase this rate, it is necessary to carry out aflatoxin biodegradation studies of these supernatants by adding ions that are thought to act as cofactors at different pH and temperatures. While the *Z. rouxii* HBB542 culture achieved 5% AFB1 degradation, no degradation occurred with the supernatant. The 5% decrease in *Z. rouxii* HBB542 culture can be explained by aflatoxin binding. There was no aflatoxin decrease in the *Z. rouxii* HBB542 supernatant, and the decrease in the culture medium can be explained by a binding mechanism rather than a degradation mechanism. Similar results were obtained in studies on *Z. rouxii* (Zhou et al. 2017). The absorption of aflatoxin by yeast depends on cell wall structures (mannan oligosaccharide structure) and physical conditions. Studies have shown that yeast protoplasts cannot reduce aflatoxin and yeast cells (*Saccharomyces* sp.) bind more AFB1 due to changes in cell wall structures after high heat/alkanity treatments (Aazami et al. 2018; Guan et al. 2021; Papp et al. 2021). It is predicted that the aflatoxin reduction potential of *Z. rouxii* HBB542 will increase with different heat and chemical treatments. This study is the first report on aflatoxin biodegradation for *Zygosaccharomyces pseudorouxii*. Optimization studies are needed in future processes for aflatoxin biodegradation with yeast isolates.

This research is the inaugural investigation of aflatoxin biodegradation in desiccated figs. In general, aflatoxin biodegradation studies are carried out by applying microorganisms to pure aflatoxin produced from *A. flavus* and degradation products can be easily seen. Since aflatoxin-containing dried fig matrix was used in the QTOF-LC/MS analyses, many ions originating from the matrix were also detected with the same analyses. Therefore, an ion library was created from previous studies in which aflatoxin degradation products were detected and these ions were searched in our study. In our study results, aflatoxin D1 and C_16_H_17_O_6_ (m/z 305.15) were detected in all biodegradation reactions where microorganism cultures were used. Aflatoxin D1 was also detected in different biodegradation studies (Chen et al., 2015; Eshelli et al. 2015). It is also known that aflatoxin D1 is less toxic than aflatoxin B1. The molecules C_12_H_23_N_2_O_2_ (m/z 227.15) and C_16_H_15_O_7_ (m/z 319.12) that we identified in the supernatant biodegradation were detected as a result of UV and ozone applications in previous studies (Mao et al. 2016; Afsah-Hejri et al. 2020). In this study, biological degradation was also reported. Although HPLC biodegradation data were low in studies conducted with yeast cultures, many ions were detected in the measurement results made with QTOF-LC/MS. When these results were examined, it was thought that longer incubation times were needed for aflatoxin biodegradation of yeasts. When the ion data and HPLC results in the negative control groups were examined, it was proven that the adapted microorganisms isolated from dried figs with aflatoxin could work synergistically and that successful results would be obtained by becoming more dominant when the selected strains were added to tturkeyhe medium.

In Türkiye, dried figs containing aflatoxin above legal limits are destroyed by burning. It is possible to use these figs as raw materials in sectors other than food by reducing or eliminating aflatoxin through biodegradation. Dried figs can be used in alcohol fermentation due to their high sugar content. In a study comparing alcohol production efficiency from dried figs with and without aflatoxin, no difference was found in terms of alcohol production efficiency at the end of the twenty-day fermentation period, and only a rapid increase in alcohol production was observed in the first 5 days of the trial conducted with figs with aflatoxin (Senturk and Karaca 2022). In our study, total sugar analyses were performed to determine whether dried figs with aflatoxin were suitable for use in ethyl alcohol production after biodegradation. According to the analysis results, sugar rates decreased by 25% as a result of biodegradation. This decrease shows that alcohol production can be higher with sugar rates before biodegradation.

This study is the first report written on aflatoxin biodegradation in dried figs containing aflatoxin. To date, a pure aflatoxin standard has been utilized in aflatoxin biodegradation. In this study, matrix effect in aflatoxin biodegradation was examined and it was shown that different results could be obtained with experiments on standard aflatoxin. Since aflatoxins develop on food products or feeds, examination of matrix effect is important for the transformation of aflatoxin. Furthermore, the total aflatoxin limitations in dried figs in Türkiye are as significant as the legal limits for aflatoxin B1 in the presence of aflatoxin. Therefore, degradation of not only aflatoxin B1 but also aflatoxin G1 is important in preventing the destruction of dried figs. Biodegradation of aflatoxins together with the food on which they are found is important in terms of preventing the destruction of these foods and showing how they can be recycled and used in different sectors.

The data obtained here can be developed in further studies to increase the aflatoxin degradation rate and shorten aflatoxin degradation times. Using recombinant technologies, aflatoxin degradation gene regions of microorganisms can be transferred to different microorganisms to make aflatoxin degradation more efficient. It is possible to determine the enzyme profiles of microorganisms that can degrade aflatoxin and to develop commercial kits by purifying these enzymes.

## Data Availability

No datasets were generated or analysed during the current study.

## References

[CR1] Aazami MH, Nasri MHF, Mojtahedi M, Mohammadi SR (2018) In vitro aflatoxin B1 binding by the cell wall and (1→ 3)-β-D-glucan of baker’s yeast. J Food Prot 81(4):670–676 29543529 10.4315/0362-028X.JFP-17-412

[CR2] Abdel-Shafi S, Shehata S, Shindia A, El-Meligy K, Khidr A (2018) Biodegradation of aflatoxins by bacteria. Egypt J Microbiol 53(1):241–254. 10.1080/10408398.2015.1106440

[CR37] abXia X, Zhang Y, Li M et al (2017) Isolation and characterization of a Bacillus subtilis strain with aflatoxin B1 biodegradation capability. Food Control 75:92–98. 10.1016/j.foodcont.2016.12.036

[CR3] Adebo OA, Njobeh PB, Gbashi S et al (2017) Review on microbial degradation of aflatoxins. Crit Rev Food Sci Nutr 57(15):3208–3217. 10.1080/10408398.2015.1106440 26517507 10.1080/10408398.2015.1106440

[CR5] Afsah-Hejri L, Hajeb P, Ehsani RJ (2020) Application of Ozone for degradation of Mycotoxins in food: A review. Compr Rev Food Sci Food Saf 19(4):1777–1808 33337096 10.1111/1541-4337.12594

[CR41] Afsharmanesh H, Perez-Garcia A, Zeriouh H, Ahmadzadeh M, Romero D (2018) Aflatoxin degradation by Bacillus subtilis UTB1 is based on production of an oxidoreductase involved in bacilysin biosynthesis. Food Control 94:48–55. 10.1016/j.foodcont.2018.03.002

[CR4] Ahmad MM, Qamar F, Saifi M, Abdin MZ (2022) Natural inhibitors: A sustainable way to combat aflatoxins. Front Microbiol 13:993834 36569081 10.3389/fmicb.2022.993834 PMC9773886

[CR7] Cai M, Qian Y, Chen N et al (2020) Detoxification of aflatoxin B1 by Stenotrophomonas sp. CW117 and characterization the thermophilic degradation process. Environ Pollut 261:114178. 10.1016/j.envpol.2020.114178 32097790 10.1016/j.envpol.2020.114178

[CR9] Chen Y, Kong Q, Chi C, Shan S, Guan B (2015) Biotransformation of aflatoxin B1 and aflatoxin G1 in peanut meal by anaerobic solid fermentation of Streptococcus thermophilus and Lactobacillus delbrueckii subsp. Bulgaricus. Int J Food Microbiol 211:1–5. 10.1016/j.ijfoodmicro.2015.06.021 26143229 10.1016/j.ijfoodmicro.2015.06.021

[CR8] Chen Y, Kong Q, Liang Y (2019) Three newly identified peptides from Bacillus megaterium strongly inhibit the growth and aflatoxin B1 production of Aspergillus flavus. Food Control 95:41–49. 10.1016/j.foodcont.2018.07.040

[CR10] Einloft TC, de Oliveira PB, Radünz LL, Dionello RG (2021) Biocontrol capabilities of three Bacillus isolates towards aflatoxin B1 producer A. flavus in vitro and on maize grains. Food Control 125:107978. 10.1016/j.foodcont.2021.107978

[CR11] Ellouze M, Buss Da Silva N, Rouzeau-Szynalski K et al (2021) Modeling B. cereus growth and cereulide formation in cereal-, dairy-, meat-, vegetable-based food and culture medium. Front Microbiol 12:639546. 10.3389/fmicb.2021.639546 33679675 10.3389/fmicb.2021.639546 PMC7925994

[CR12] Eshelli M, Harvey L, Edrada-Ebel R, McNeil B (2015) Metabolomics of the bio-degradation process of aflatoxin B1 by actinomycetes at an initial pH of 6.0. Toxins 7(2):439–456. 10.3390/toxins7020439 25658510 10.3390/toxins7020439 PMC4344634

[CR42] Fang QA, Du M, Chen J, Liu T, Zheng Y, Liao Z, Wang J (2020) Degradation and detoxification of aflatoxin B1 by tea-derived Aspergillus niger RAF106. Toxins 12(12):777. 10.3390/toxins12120777 10.3390/toxins12120777 PMC7762301 33291337

[CR13] Fletcher MT, Blaney BJ (2016) Mycotoxins. Reference Module in Food Science

[CR14] Goswami G, Panda D, Samanta R, Boro RC, Modi MK, Bujarbaruah KM, Barooah M (2018) Bacillus megaterium adapts to acid stress condition through a network of genes: insight from a genome-wide transcriptome analysis. Sci Rep 8(1):16105. 10.1038/s41598-018-34221-0 30382109 10.1038/s41598-018-34221-0 PMC6208408

[CR15] Guan S, Ji C, Zhou T, Li J, Ma Q, Niu T (2008) Aflatoxin B1 degradation by Stenotrophomonas maltophilia and other microbes selected using coumarin medium. Int J Mol Sci 9(8):1489–1503. 10.3390/ijms9081489 19325817 10.3390/ijms9081489 PMC2635738

[CR16] Guan Y, Chen J, Nepovimova E et al (2021) Aflatoxin detoxification using microorganisms and enzymes. Toxins 13(1):46. 10.3390/toxins13010046 33435382 10.3390/toxins13010046 PMC7827145

[CR17] Hwang HH, Chien PR, Huang FC et al (2022) A plant endophytic bacterium P. megaterium StrainBP-R2 isolated from the halophyte Bolboschoenus planiculmis enhances plant growth under salt and drought stresses. Microorganisms 10(10):2047. 10.3390/microorganisms10102047 10.3390/microorganisms10102047 PMC9610499 36296323

[CR18] Javanmardi F, Khodaei D, Sheidaei Z, Bashiry M et al (2020) Decontamination of aflatoxins in edible oils: A comprehensive review. Food Reviews Int 38(7):1410–1426. 10.1080/87559129.2020.1812635

[CR40] Karaca H, Velioglu YS, Nas S (2010) Mycotoxins: contamination of dried fruits and degradation by ozone. Toxin Rev 29(2):51–59. 10.3109/15569543.2010.485714

[CR19] Le Marc Y, Baert L, Da Silva NB et al (2021) The effect of pH on the growth rate of B. cereus sensu lato: quantifying strain variability and modelling the combined effects of temperature and pH. Int J Food Microbiol 360:109420. 10.1016/j.ijfoodmicro.2021.109420 34602293 10.1016/j.ijfoodmicro.2021.109420

[CR20] Liang H, Lin F, Zhang Z et al (2017) Multicopper laccase mimicking nanozymes with nucleotides as ligands. ACS Appl Mater Interfaces 9(2):1352–1360 28004568 10.1021/acsami.6b15124

[CR43] Liu Y, Mao H, Yohannes KW, Wan Z, Cao Y, Tron T, Wang J (2021) Degradation of aflatoxin B1 by a recombinant laccase from Trametes sp. C30 expressed in Saccharomyces cerevisiae: A mechanism assessment study in vitro and in vivo. Food Res Int 145:110418. 10.1016/j.foodres.2021.110418 10.1016/j.foodres.2021.110418 34112421

[CR21] Loi M, Fanelli F, Liuzzi V (2017) Mycotoxin Biotransformation by Native and Commercial Enzymes: Present and Future Perspectives. Toxins, 9(4), 111. MDPI AG. Retrieved from 10.3390/toxins9040111 10.3390/toxins9040111 PMC5408185 28338601

[CR22] Luo Y, Liu X, Yuan L, Li J (2020) Complicated interactions between bio-adsorbents and Mycotoxins during Mycotoxin adsorption: current research and future prospects. Trends Food Sci Technol 96:127–134. 10.1016/j.tifs.2019.12.012

[CR23] Mahariawan IMD, Kusuma WE, Yuniarti A et al (2020) Effect of temperature and pH combination on vegetative cell growth of Bacillus megaterium. In Journal of Physics: Conference Series (Vol. 1665, No. 1, p. 012013). IOP Publishing. 10.1088/1742-6596/1665/1/012013

[CR24] Mantea LE, El-Sabeh A, Mihasan M et al (2025) B. safensis P1. 5S exhibits Phosphorus-Solubilizing activity under abiotic stress. Horticulturae 11(4):388. 10.3390/horticulturae11040388

[CR25] Mao J, He B, Zhang L (2016) A structure identification and toxicity assessment of the degradation products of aflatoxin B1 in peanut oil under UV irradiation. Toxins 8(11):332 27845743 10.3390/toxins8110332 PMC5127128

[CR26] Nazilli Chamber of Commerce Dried Fig Report (2019) https://www.naztic.org.tr/wp-content/uploads/2021/03/kuru-incir-sektor-raporu.pdf. Son Erişim:16.09.2021

[CR27] Papp LA, Horváth E, Peles F et al (2021) Insight into yeast–mycotoxin relations. Agriculture 11(12):1291. 10.3390/agriculture11121291

[CR44] Pereyra MG, Martínez MP, Cavaglieri LR (2019) Presence of aiiA homologue genes encoding for N-Acyl homoserine lactone-degrading enzyme in aflatoxin B1-decontaminating Bacillus strains with potential use as feed additives. Food Chem Toxicol 124:316–323. 10.1016/j.fct.2018.12.016 10.1016/j.fct.2018.12.016 30557671

[CR28] Prettl Z, Dési E, Lepossa A, Kriszt B, Kukolya J, Nagy E (2017) Biological degradation of aflatoxin B 1 by a Rhodococcus pyridinivorans strain in by-product of bioethanol. Anim Feed Sci Technol 224:104–114. 10.1016/j.anifeedsci.2016.12.011

[CR46] Rao KR, Vipin AV, Hariprasad P, Appaiah KA, Venkateswaran GJFC (2017) Biological detoxification of Aflatoxin B1 by Bacillus licheniformis CFR1. Food Control 71:234–241. 10.1016/j.foodcont.2016.06.040

[CR45] Ren X, Zhang Q, Zhang W, Mao J, Li P (2020) Control of aflatoxigenic molds by antagonistic microorganisms: Inhibitory behaviors, bioactive compounds, related mechanisms, and influencing factors. Toxins 12(1):24. 10.3390/toxins12010024 10.3390/toxins12010024 PMC7020460 31906282

[CR30] Senturk S, Karaca H (2022) First report on the presence of aflatoxins in Fig seed oil and the efficacy of adsorbents in reducing aflatoxin levels in aqueous and oily media. Toxin Reviews 41(3):817–827. 10.1080/15569543.2021.1937226

[CR31] Shu X, Wang Y, Zhou Q et al (2018) Biological degradation of aflatoxin B1 by cell-free extracts of Bacillus velezensis DY3108 with broad pH stability and excellent thermostability. Toxins 10(8):330. 10.3390/toxins10080330 30110983 10.3390/toxins10080330 PMC6116002

[CR32] Song C, Yang J, Wang Y (2022) Mechanisms and transformed products of aflatoxin B1 degradation under multiple treatments: a review. Crit Rev Food Sci Nutr 1–13. 10.1080/10408398.2022.2121910 10.1080/10408398.2022.2121910 36102160

[CR33] Suresh G, Cabezudo I, Pulicharla R (2020) Biodegradation of aflatoxin B1 with cell-free extracts of trametes versicolor and Bacillus subtilis. Res Vet Sci 133:85–91. 10.1016/j.rvsc.2020.09.009 32957062 10.1016/j.rvsc.2020.09.009

[CR34] Verheecke C, Liboz T, Mathieu F (2016) Microbial degradation of aflatoxin B1: current status and future advances. Int J Food Microbiol 237:1–9. 10.1016/j.ijfoodmicro.2016.07.028 27541976 10.1016/j.ijfoodmicro.2016.07.028

[CR35] Wang J, Xie Y (2020) Review on microbial degradation of Zearalenone and aflatoxins. Grain Oil Sci Technol 3(3):117–125. 10.1016/j.gaost.2020.05.002

[CR36] Wang L, Huang W, Shen Y (2022) Enhancing the degradation of aflatoxin B1 by co-cultivation of two fungi strains with the improved production of detoxifying enzymes. Food Chem 371:131092. 10.1016/j.foodchem.2021.131092 34543924 10.1016/j.foodchem.2021.131092

[CR38] Xiong D, Wen J, Lu G et al (2022) Isolation, purification, and characterization of a laccase-degrading aflatoxin B1 from Bacillus amyloliquefaciens B10. Toxins 14(4):250. 10.3390/toxins14040250 35448859 10.3390/toxins14040250 PMC9028405

[CR39] Zhao C, Dong L, Zhang F et al (2022) Screening and characterization of a salt-tolerant aflatoxin B 1-degrading strain isolated from doubanjiang, a Chinese typical red pepper paste. Food Science and Technology, p 42

[CR47] Zhou G, Chen Y, Kong Q, Ma Y, Liu Y (2017) Detoxification of aflatoxin B1 by Zygosaccharomyces rouxii with solid state fermentation in peanut meal. Toxins 9(1):42. 10.3390/toxins9010042 10.3390/toxins9010042 PMC5308274 28117705

